# Combined oral intake of short and long fructans alters the gut microbiota in food allergy model mice and contributes to food allergy prevention

**DOI:** 10.1186/s12866-023-03021-6

**Published:** 2023-09-22

**Authors:** Hideaki Takahashi, Tadashi Fujii, Saki Yamakawa, Chikako Yamada, Kotoyo Fujiki, Nobuhiro Kondo, Kohei Funasaka, Yoshiki Hirooka, Takumi Tochio

**Affiliations:** 1 Graduate School of Nutritional Sciences, Nagoya University of Arts and Sciences, Nisshin, Aichi Japan; 2 Department of Gastroenterology and Hepatology, Fujita Health University, Toyoake, Aichi Japan; 3 Department of Medical Research on Prebiotics and Probiotics, Fujita Health University, Toyoake, Aichi Japan; 4 Research and Development Division, Itochu Sugar Co., Ltd., Hekinan, Aichi Japan; 5 WELLNEO SUGAR Co., Ltd., Tokyo, Japan

**Keywords:** Fructan, Food allergy, Microbiota, *Parabacteroides*, Short-chain fatty acids

## Abstract

**Background:**

It has become clear that the intestinal microbiota plays a role in food allergies. The objective of this study was to assess the food allergy-preventive effects of combined intake of a short fructan (1-kestose [Kes]) and a long fructan (inulin ([Inu]) in an ovalbumin (OVA)-induced food allergy mouse model.

**Results:**

Oral administration of fructans lowered the allergenic symptom score and alleviated the decreases in rectal temperature and total IgA levels and increases in OVA-specific IgE and IgA levels induced by high-dose OVA challenge, and in particular, combined intake of Kes and Inu significantly suppressed the changes in all these parameters. The expression of the pro-inflammatory cytokine IL-4, which was increased in the allergy model group, was significantly suppressed by fructan administration, and the expression of the anti-inflammatory cytokine IL-10 was significantly increased upon Kes administration. 16 S rRNA amplicon sequencing of the gut microbiota and beta diversity analysis revealed that fructan administration may induce gut microbiota resistance to food allergy sensitization, rather than returning the gut microbiota to a non-sensitized state. The relative abundances of the genera *Parabacteroides B 862,066* and *Alloprevotella*, which were significantly reduced by food allergy sensitization, were restored by fructan administration. In *Parabacteroides*, the relative abundances of *Parabacteroides distasonis*, *Parabacteroides goldsteinii*, and their fructan-degrading glycoside hydrolase family 32 gene copy numbers were increased upon Kes or Inu administration. The concentrations of short-chain fatty acids (acetate and propionate) and lactate were increased by fructan administration, especially significantly in the Kes + Inu, Kes, and Inu-fed (Inu, Kes + Inu) groups.

**Conclusion:**

Combined intake of Kes and Inu suppressed allergy scores more effectively than single intake, suggesting that Kes and Inu have different allergy-preventive mechanisms. This indicates that the combined intake of these short and long fructans may have an allergy-preventive benefit.

**Supplementary Information:**

The online version contains supplementary material available at 10.1186/s12866-023-03021-6.

## Background

Recent decades have witnessed an increase in the incidence of food allergies [1]. A food allergy is defined as “an adverse health effect arising from a specific immune response that occurs reproducibly on exposure to a given food” [2]. Food allergy is currently a considerable public health concern, affecting 3–6% of children in developed countries, and the diagnosis of food allergy negatively affects the quality of life of patients and their families and poses a significant financial burden [3, 4]. Hence, research on simple and effective methods for preventing food allergy is of great public health importance.

In recent years, it has become clear that the intestinal microbiota plays a role in food allergies. For example, analysis of the fecal microbiota of 66 egg-allergic children and 75 control children revealed microbiota dysbiosis in the egg-allergic children [5]. Feehley et al. colonized germ-free mice with feces from healthy or cow’s milk-allergic infants and found that germ-free mice colonized with bacteria from healthy, but not allergic infants were protected against anaphylactic responses to a cow’s milk allergen [6]. These studies strongly suggest a relationship between gut microbiota dysbiosis and food allergy; however, few studies have shown that improving gut microbiota health improves food allergy.

Prebiotics was defined at the 6th Meeting of the International Scientific Association of Probiotics and Prebiotics (ISAPP) in 2008 as “a selectively fermented ingredient that results in specific changes in the composition and/or activity of the gastrointestinal microbiota, thus conferring benefit(s) upon host health” [7]. There are numerous types of prebiotics. Many of them are a subset of the carbohydrate group, mostly oligosaccharide carbohydrates. Recent research reports have demonstrated that prebiotics not only improve intestinal microbiota dysbiosis but also have a positive effect on various diseases [8].

Fructans consist of a linear chain of fructose molecules linked via β(2→1) linkages. They generally have terminal glucose units linked via β(2→1) linkages. The degree of polymerization (DP) of 1-Kestose (Kes) is 3, whereas that of fructo-oligosaccharides is < 10 and that of inulin (Inu) is up to 60. Studies have long suggested that fructans selectively stimulate lactic acid bacteria [9, 10]. Recent studies have shown that other bacteria can ferment fructan depending on the fructan DP [11, 12].

Several studies have reported improvement and prevention of allergic diseases by fructans. For example, Kes exerts a beneficial effect on clinical symptoms in infants with atopic dermatitis [13], and Inu prevents the onset of allergies [14]. Based on these findings, we speculated that low-DP Kes and high-DP Inu have different mechanisms of allergy improvement and that combined intake of Kes and Inu may result in stronger food allergy improvement effects than single intake. To test this hypothesis, herein, we investigated the effects of Kes and/or Inu on food allergy prevention in an ovalbumin (OVA)-induced food allergy mouse model.

## Results

### Effects of fructans on OVA-induced food allergy markers

**Fig. 1:**
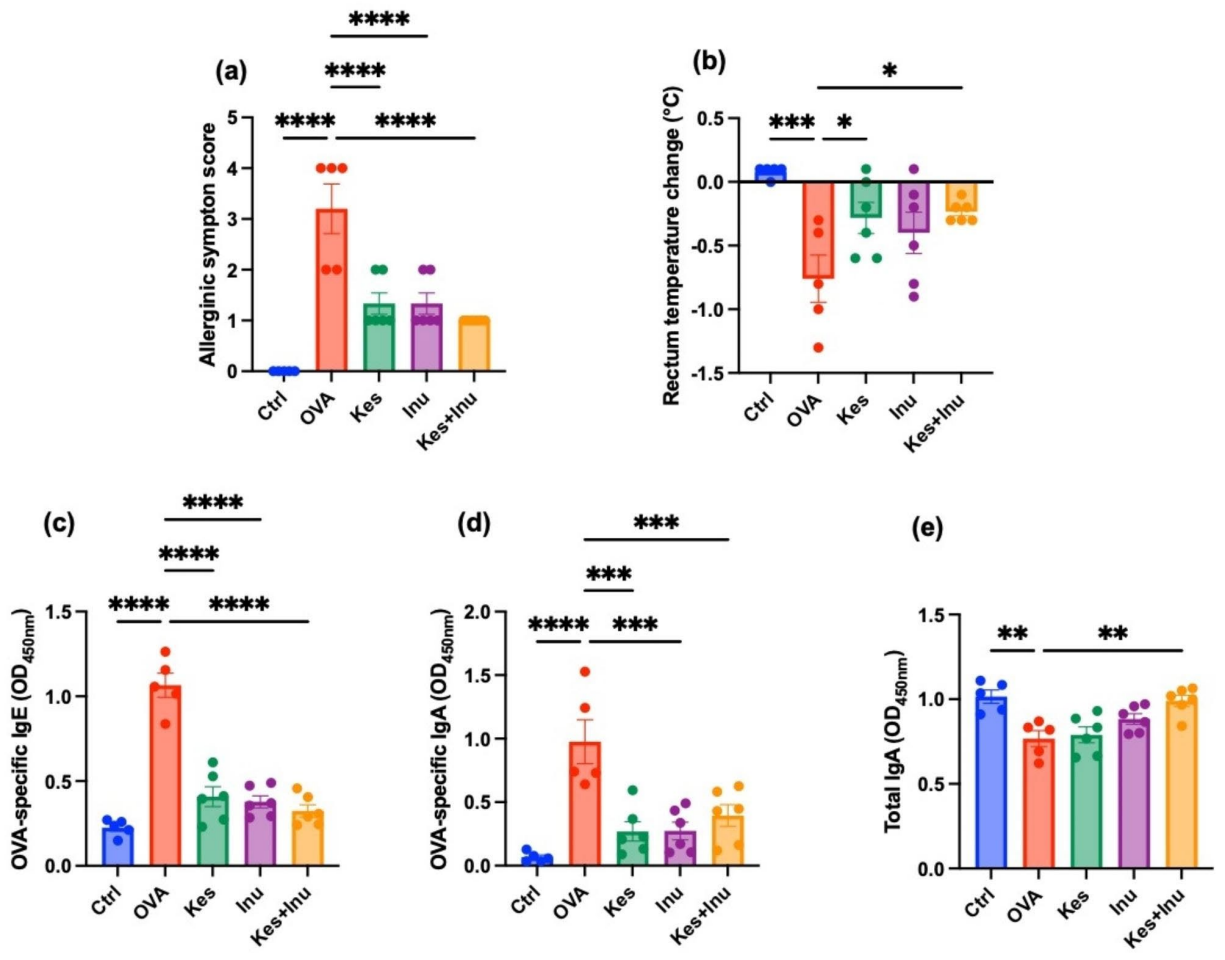
Severity of OVA-induced food allergy with and without fructan administration. (**a**) Allergy scores of BALB/c mice subjected to food allergy. (**b**) Changes in rectal temperature 30 min after oral administration of OVA. (**c**) OVA-specific IgE levels in the serum, (**d**) OVA-specific IgA levels in the feces, and (**e**) total IgA levels in the feces. Plots represent individual mice, and bars represent mean ± SEM. *P < 0.05, **P < 0.01, ***P < 0.001, ****P < 0.0001 vs. OVA group (ordinary one-way ANOVA)

We used an OVA-induced food allergy mouse model to test the anti-allergic effects of the low-DP fructan Kes and the high-DP fructan Inu. The results showed that mice in the OVA group had significantly higher allergenic symptom scores than those in the Ctrl group, whereas fructan-fed (Kes, Inu, Kes + Inu) mice had significantly lower allergenic symptom scores than those in the OVA group (Fig. 1a). Rectal temperature was significantly decreased in the OVA group, but treatment with Kes (Kes, Kes + Inu) significantly attenuated the decrease in rectal temperature in mice (Fig. 1b). No significant differences were observed in body weight or food intake between any of the groups.

To monitor the effects of the fructan treatments, OVA-specific IgE and IgA levels and total IgA levels were measured using an enzyme-linked immunosorbent assay (ELISA). Serum OVA-specific IgE and fecal OVA-specific IgA levels were significantly increased in the OVA group compared to those in the Ctrl group, but these increases were significantly suppressed by fructan administration (Kes, Inu, Kes + Inu) (Fig. 1c, d). Fecal total IgA levels were significantly decreased in the OVA group, but this decrease was significantly suppressed in the Kes + Inu group (Fig. 1e).

### Effects of fructans on cytokine gene expression in Peyer’s patches

**Fig. 2:**
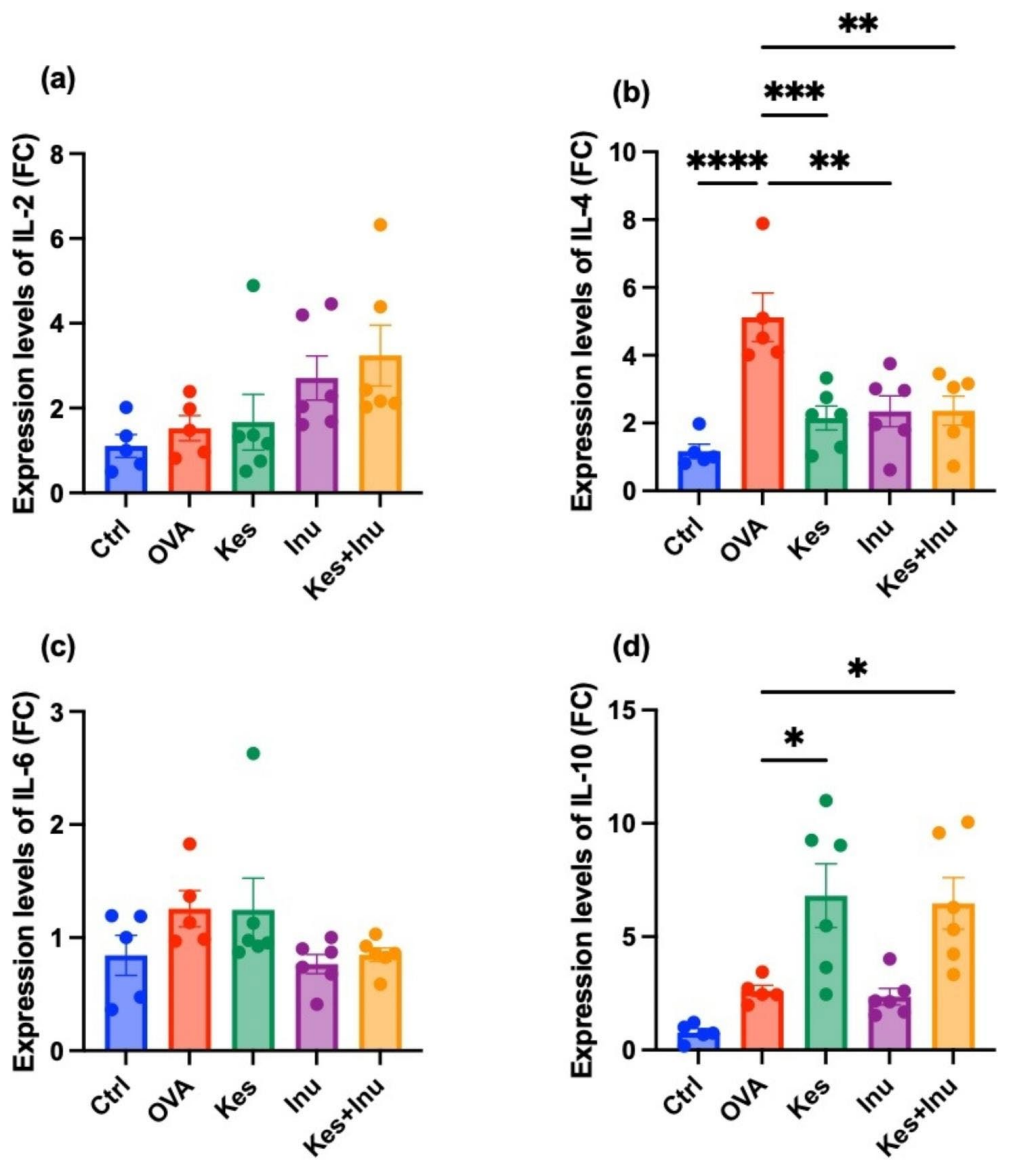
RT-qPCR assay of cytokine gene expression in OVA-induced food allergy with or without fructan administration. Gene expression levels of (**a**) IL-2, (**b**) IL-4, (**c**) IL-6, and (**d**) IL-10 in Peyer’s patches. Plots represent individual mice, and bars represent mean ± SEM. *P < 0.05, **P < 0.01, ***P < 0.001, ****P < 0.0001 vs. OVA group (ordinary one-way ANOVA)

We next examined the effects of the fructans on inflammatory cytokine expression in Peyer’s patches after OVA challenge. The expression level of the pro-inflammatory cytokine IL-4 in the OVA group was significantly increased compared with that in the Ctrl group, but this increase was significantly suppressed by fructan administration (Kes, Inu, Kes + Inu) (Fig. 2b). The expression of the anti-inflammatory cytokine IL-10 did not significantly differ between the OVA and Ctrl groups, but it was significantly increased in the Kes-fed (Kes, Kes + Inu) groups (Fig. 2d). However, the expression levels of the anti-inflammatory cytokines IL-2 and IL-6 did not significantly differ among the groups (Fig. 2a, c).

### Effects of fructans on the intestinal microbiota

**Fig. 3:**
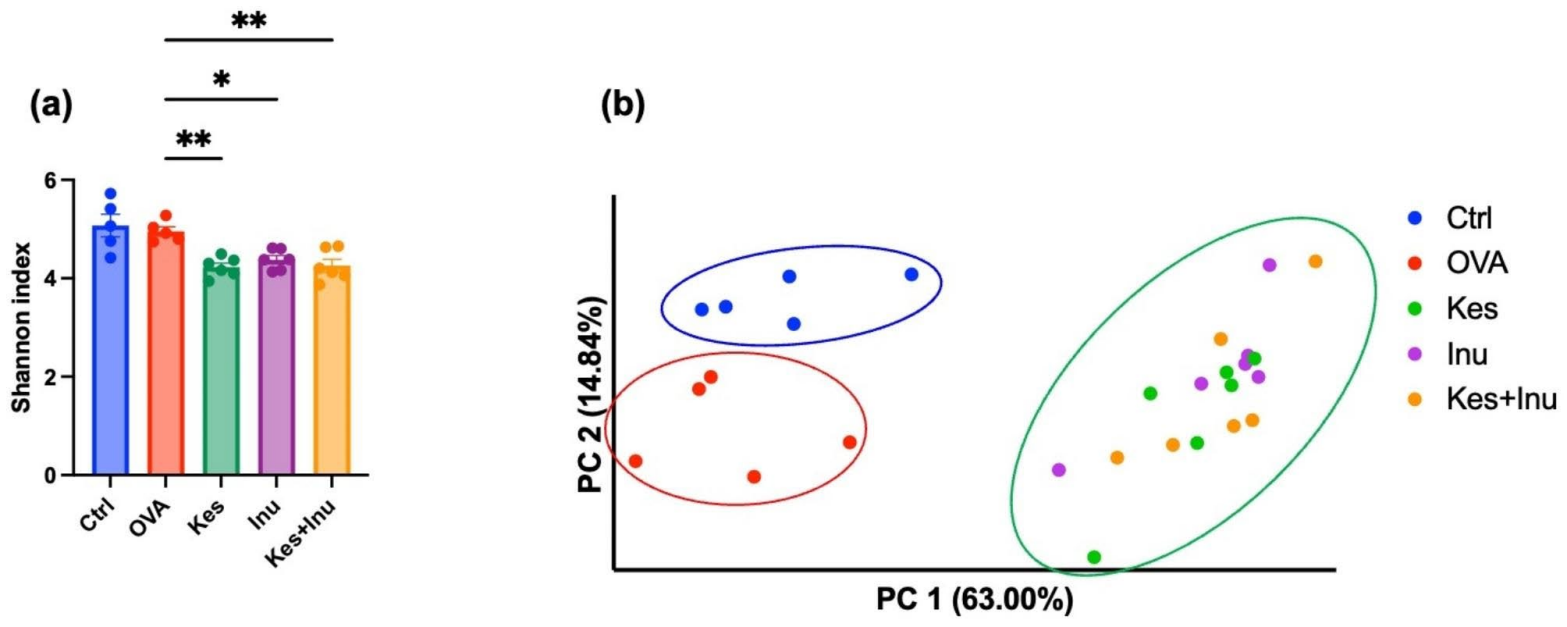
Alpha and beta diversity of the intestinal microbiota in each experimental group. (**a**) Shannon index values. *P < 0.05, **P < 0.01 vs. OVA group (ordinary one-way ANOVA). (**b**) Principal coordinate analysis based on weighted UniFrac distances. The first primary component is plotted on the horizontal axis (PC1) and the second primary component is plotted on the vertical axis (PC2). Plots represent individual mice and bars represent mean ± SEM

The microbiota has an essential influence on immune cell homeostasis [15] and susceptibility to allergic inflammation [16]. Therefore, we analyzed the effects of the fructans on the intestinal microbiota in the OVA-induced food allergy mouse model. The Shannon diversity index (alpha diversity) was calculated to measure differences in taxonomic diversity among the groups, and it was significantly decreased upon fructan administration (Fig. 3a). Similarities among all samples were evaluated via principal coordinate analysis and ecologic distances calculated based on the weighted UniFrac distance (beta diversity). The Ctrl and OVA groups were significantly separated (*P* = 0.006) (Fig. 3b). There was also a significant difference in beta diversity between the OVA group and each fructan intake group (Kes, Inu, Kes + Inu: *P* = 0.006, 0.003, 0.004, respectively) (Fig. 3b).

**Fig. 4:**
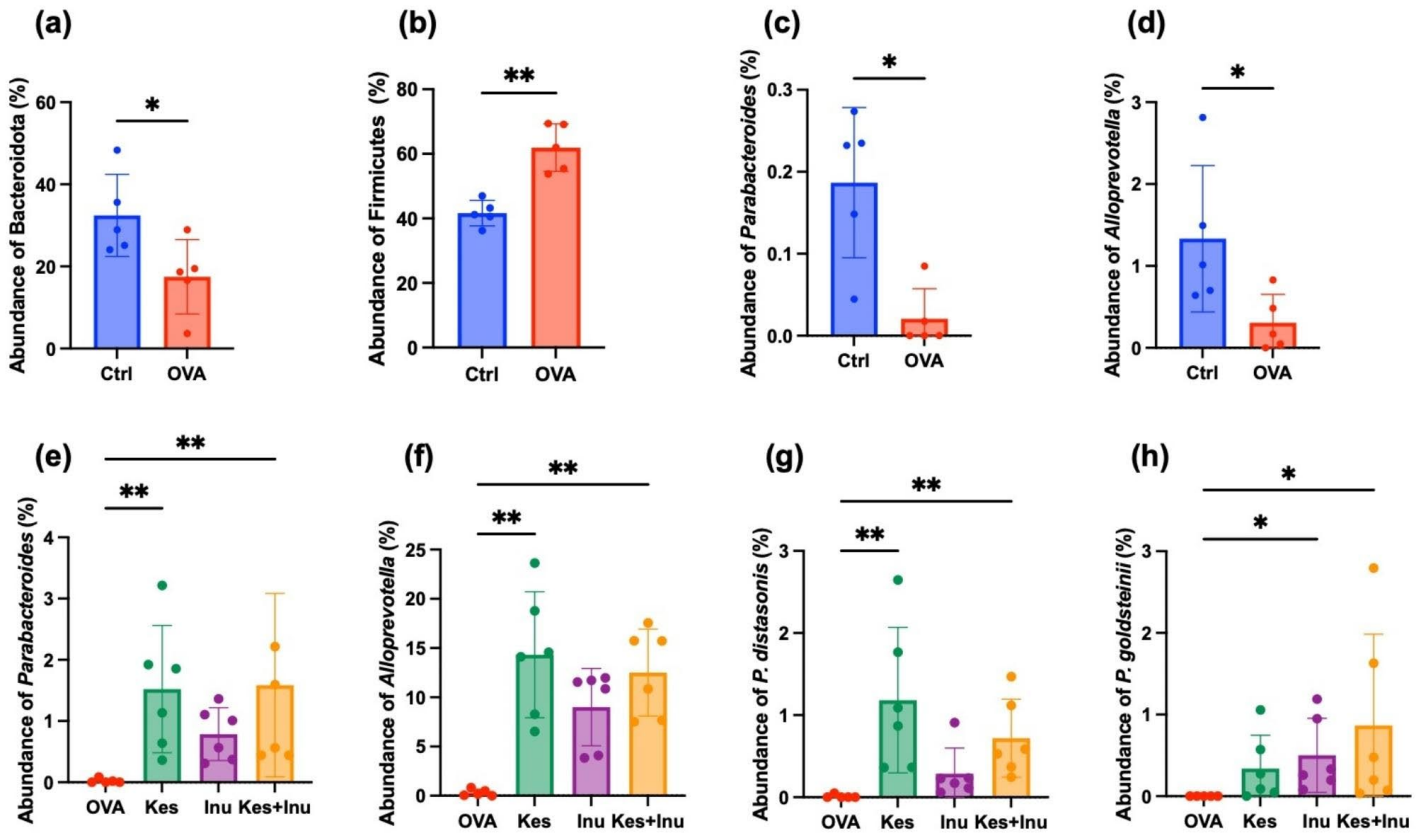
Changes in relative abundances of (**a**) Bacteroidota, (**b**) Firmicutes, (**c**) *Parabacteroides*, and (**d**) *Alloprevotella* in OVA-induced food allergic mice. Statistical significance is determined using the Mann–Whitney test. Fructan administration suppresses the reductions in the relative abundances of (**e**) *Parabacteroides*, (**f**) *Alloprevotella*, (**g**) *P. distasonis*, and (**h**) *P. goldsteinii* induced by OVA-induced food allergy. Plots represent individual mice, and bars represent mean ± SEM. *P < 0.05, **P < 0.01, ***P < 0.001 vs. OVA group (Kruskal–Wallis test)

Analysis of the relative abundances of intestinal bacteria at the phylum level revealed that only Bacteroidota were significantly less abundant and only Firmicutes were significantly more abundant in the OVA group compared to the Ctrl group (Fig. 4a, b). A detailed genus-level analysis of Bacteroidota revealed that mice in the OVA group had significantly reduced relative abundances of the genera *Parabacteroides B 862,066* (*Parabacteroides*) and *Alloprevotella* compared to the Ctrl group (Fig. 4c, d). These reductions were restored in the fructan-fed groups, especially in the Kes-fed (Kes, Kes + Inu) groups, which showed significant restoration of the relative abundances of the genera (Fig. 4e, f). Further detailed analysis revealed that only three amplicon sequence variants (ASVs) were classified in the genus *Parabacteroides*, with an ASV abundances of 45.1%, 53.3%, and 1.6% (data not shown). The ASVs with 45.1% abundance were found to be from *Parabacteroides distasonis* and those with 53.3% abundance were from *Parabacteroides goldsteinii*. The relative abundances of *P. distasonis* and *P. goldsteinii* were significantly higher in the Kes-fed and Inu-fed groups, respectively, than in the OVA group (Fig. 4g, h). The bacterial species constituting *Alloprevotella* could not be identified.

### Effects of fructans on GH32 gene copy numbers in fecal samples and the fructan-degrading activity of GH32 enzymes

We tested the hypothesis that the significant increases in the abundances of *P. distasonis* and *P. goldsteinii* in the Kes and Inu groups, respectively, were due to the fact that each species has a gene encoding an enzyme that degrades Kes and Inu, respectively. Fructans are generally hydrolyzed by glycoside hydrolase (GH) family 32 enzymes, and the resultant fructose and glucose are metabolized through indigenous metabolic pathways in each microbial species [17].

**Fig. 5:**
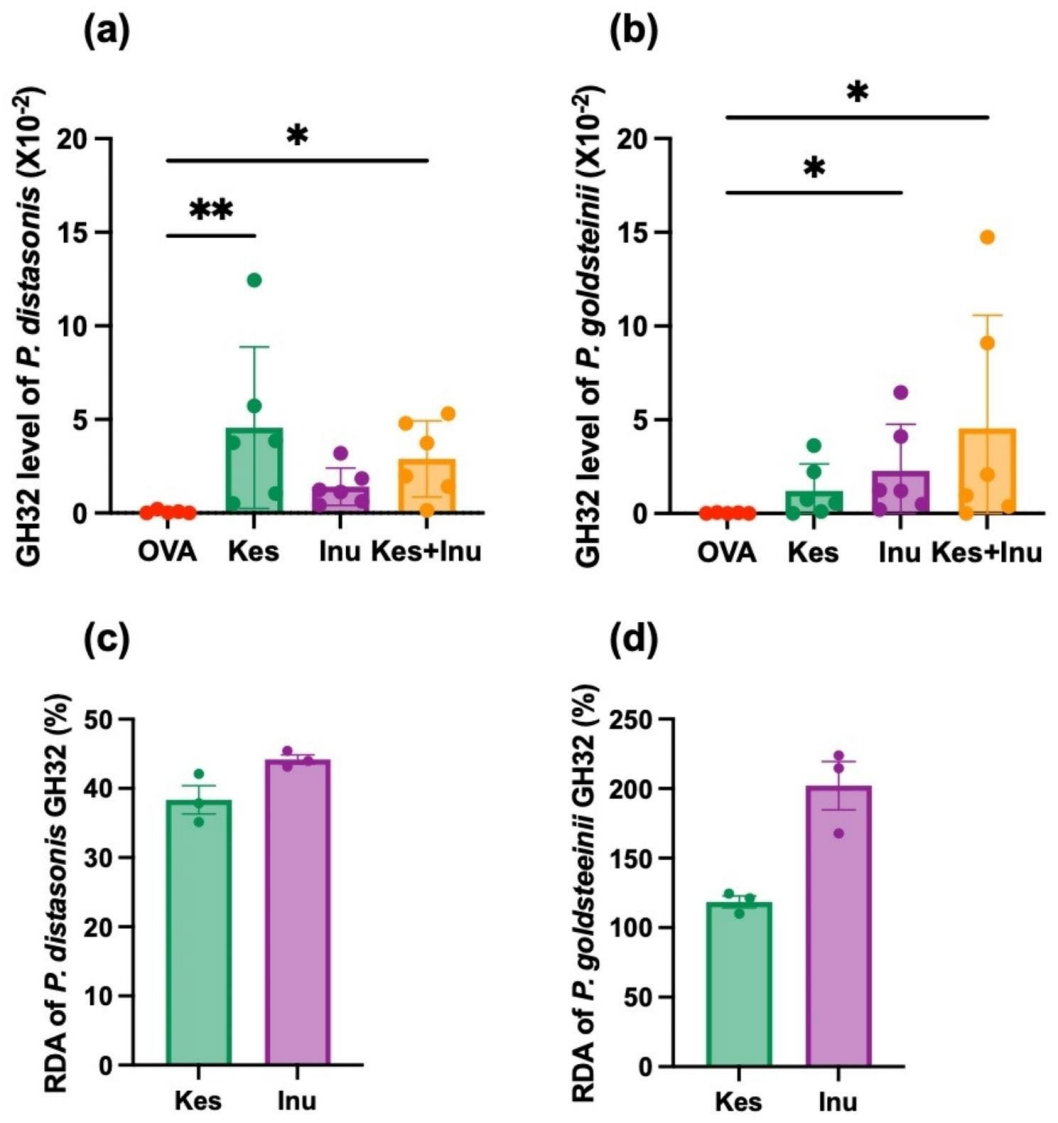
GH32 gene copy numbers in (**a**) *P. distasonis* and (**b**) *P. goldsteinii* in fecal samples. Circles represent individual mice, and bars represent mean ± SEM. *P < 0.05, **P < 0.01 vs. OVA group (Kruskal–Wallis test). Relative degradation activities of recombinant GH32 enzymes of *P. distasonis* (**c**) and *P. goldsteinii* (**d**) towards Kes and Inu against sucrose. Plots represent individual mice, and bars represent mean ± SEM

First, we designed a primer set based on the consensus sequence of the GH32 genes of *P. distasonis* and *P. goldsteinii.* Quantitative (q)PCR analysis using this primer set revealed that GH32 gene copy numbers in *P. distasonis* and *P. goldsteinii* in fecal samples were significantly increased in the Kes-fed and Inu-fed groups, respectively (Fig. 5a, b), which was in line with the relative abundance of each species.

Next, we determined the fructan-degrading activity of the GH32 enzymes of *P. distasonis* and *P. goldsteinii.* As GH32 enzymes of these species are extracellular enzymes and are difficult to express intracellularly, they were expressed on *Escherichia coli* cells using a surface display system [18], and whole cells of transformed *E. coli* were used to assess the activities of the GH32 enzymes on sucrose, Kes, and Inu. The *P. distasonis* GH32 gene fragment amplified from fecal samples of mice in the Kes group (GenBank accession No. LC771243) showed 99.18% identity with the GH32 gene of *P. distasonis* strain BFG-592 and other strains. The *P. goldsteinii* GH32 gene fragment amplified from fecal samples of mice in the Inu group (GenBank accession No. LC771244) showed 99.89% identity with the GH32 gene of *P. goldsteinii* strain MTS01. The results of the enzymatic reaction assays using *E. coli* expressing GH32 from *P. distasonis* and *P. goldsteinii* confirmed that the enzymes had Kes- and Inu-degrading activity (Fig. 5c, d). In a test using *E. coli* harboring pCDF-PgsA as a negative control, no fructose due to substrate degradation was detected.

### Effects of fructans on intestinal short-chain fatty acid (SCFA) contents

**Fig. 6:**
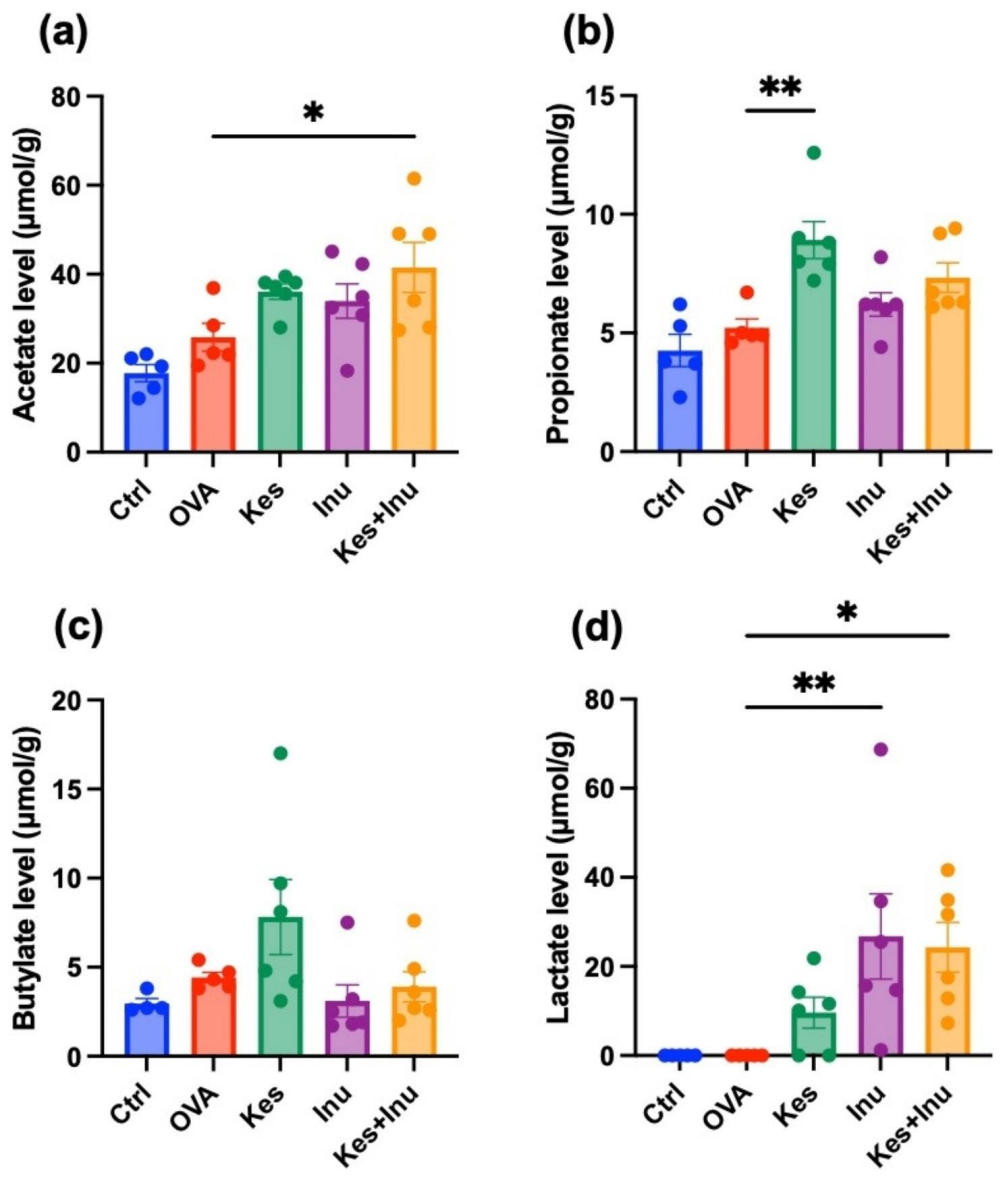
Changes in (**a**) acetate, (**b**) propionate, (**c**) n-butyrate, and (**d**) lactate levels in cecal samples of OVA-induced food allergic mice with and without fructan administration. Plots represent individual mice, and bars represent mean ± SEM. *P < 0.05, **P < 0.01 vs. OVA group (ordinary one-way ANOVA)

Commensal bacteria metabolize fibers and generate SCFAs (acetate, propionate, and butylate), which stimulate regulatory T (Treg) cell expansion and immune-suppressive properties, such as IL-10 production, thereby controlling proinflammatory responses in the gut [19]. We determined the concentrations of SCFAs (acetate, propionate, n-butyrate) and lactate in the cecal contents of mice. Compared to the OVA group, the concentrations of acetate, propionate, and lactate were increased in the fructan-fed groups, with significant increases in acetate in Kes + Inu group, propionate in Kes group, and lactic acid in Inu-fed (Inu, Kes + Inu) group (Fig. 6). However, the fructans did not significantly affect the cecal butyrate concentration.

## Discussion

Previous studies have suggested that the low-DP Kes and the high-DP Inu have different allergy-preventive mechanisms, and that combined intake of Kes and Inu may exert an effective food allergy-preventive effect. The objective of this study was to determine the food allergy-preventive effects of combined intake of Kes and Inu using an OVA-allergic mouse model.

Oral administration of fructans lowered the allergenic symptom score and suppressed the decreases in rectal temperature and total IgA levels and the increases in OVA-specific IgE and IgA levels induced by high-dose OVA challenge, indicating that fructan administration suppressed typical allergic symptoms in the mouse model. In particular, combined intake of Kes and Inu significantly and most effectively suppressed in all these parameters, suggesting that it may be effective in controlling food allergy symptoms.

Peyer’s patches are a component of gut-associated lymphoid tissue and are involved in the regulation of immune responses to intestinal substances, such as bacteria. Cytokines expressed in Peyer’s patches reportedly are involved in food allergy symptoms [20]. Therefore, we examined the mRNA expression levels of various cytokines associated with food allergy in Peyer’s patches. The gene expression levels of IL-2, produced by Th1 cells and involved in cellular immunity by stimulating macrophages, and IL-6, involved in both immune responses and inflammation, were not significantly different among the treatment groups. The gene expression level of IL-4, produced by Th2 cells and involved in humoral immunity by activating B cells, was increased in the allergic group, but significantly suppressed by fructan administration. These results suggest that ingestion of both short and long fructans has an allergy-preventive effect by modulating immune responses involving IL-4 or Th2 cells. In contrast, the expression level of IL-10, an anti-inflammatory cytokine produced by Th1 cells, did not significantly differ between the OVA and Ctrl groups but was significantly increased by Kes administration (not Inu), suggesting that intake of short fructans may have an allergy-preventive effect by modulating immune responses involving IL-10 or Th1 cells. However, we only examined the mRNA levels of cytokines in Peyer’s patches, and protein measurements using ELISA and cell-level measurements using flow cytometry to examine the detailed effects of cytokines on immune cells should be conducted in future.

Allergic diseases have been linked to environmental and lifestyle changes driving the dysfunction of three interdependent biological systems: the microbiota, epithelial barrier, and immune system [12]. We speculated that the differences in the preventive effects of Kes and Inu on OVA-induced allergy may be due to differences in their effects on the gut microbiota; therefore, we performed 16 S rRNA amplicon sequencing of the gut microbiota.　Bioinformatics analysis revealed no significant change in alpha diversity in the OVA group compared to the Ctrl group but showed a clear difference in beta diversity. Despite the fact that intraperitoneal rather than intestinal administration of allergens, these results suggested that the intestinal microbiota may have been affected. In the fructan-fed group, alpha diversity was significantly reduced, and beta diversity was clearly distinct from both the Ctrl and OVA groups. This suggests that fructan administration may induce gut microbiota resistance to food allergy sensitization, rather than returning the gut microbiota to a non-sensitized state. In the present study, we did not analyze the baseline gut microbiota before the introduction of the different diets, just before the first OVA injection, and before the last oral dose of OVA. However, no difference in the gut microbiota is expected before the introduction of the different diets because the mice were pre-fed for 1 week before being fed the different diets.

As fructans can restore some intestinal bacterial growth, the present study focused on Bacteroidota, the only phylum that was significantly reduced by OVA sensitization. In Bacteroidota, the genera *Parabacteroides* and *Alloprevotella* were significantly lower in abundance in the OVA group. These decreases were restored in the fructan-fed group, especially significantly in the Kes-fed group. In this study, *P. distasonis* and *P. goldsteinii* constituted the genus *Parabacteroides*, and these species showed significantly increased relative abundances after the administration of Kes or Inu, respectively. *Alloprevotella* has been reported to be a SCFA-producing bacterium, and its abundance was positively correlated with the IL-10 level in a mouse model of dextran sodium sulfate-induced colitis [21]. *P. distasonis* suppressed IL-4 levels and induced IL-10 levels in the colonic mucosa of azoxymethane-treated mice [22], and primed dendritic cells to induce Treg cells from naïve CD4^+^ T cells and induced IL-10 production by peripheral blood mononuclear cells [23]. *P. goldsteinii* promotes the production of Treg cells and IL-10 and improves prediabetes syndromes and liver inflammation [24]. The results of this study—and of a few previous studies—suggest that *Alloprevotella*, *P. distasonis*, and *P. goldsteinii*, which are increased by fructan administration, likely have anti-inflammatory and anti-allergic effects.

The results of qPCR targeting the GH32 genes of *P. distasonis* and *P. goldsteinii* showed that GH32 gene copy numbers in *P. distasonis* and *P. goldsteinii* were significantly increased in the Kes-fed and Inu-fed groups, respectively, which was in line with the relative abundances of these species. Moreover, enzymatic reaction assays using *E. coli* expressing GH32 from *P. distasonis* and *P. goldsteinii* confirmed that the enzymes had Kes- and Inu-degrading activity. The DP of fructan that a GH32 enzyme can degrade differs depending on the enzyme, and many GH32 enzymes that can only degrade sucrose [25]. These results suggest that the relative abundances of *P. distasonis* and *P. goldsteinii* may have increased due to Kes and/or Inu administration because they harbor a gene encoding GH32 that degrades Kes and/or Inu. From the perspective of enzyme engineering, the present results are interesting because there have been reports on fructan metabolism by GH32 enzymes of bifidobacteria and butyrate-producing bacteria [25, 26], but the GH32 enzymes of *Parabacteroides* spp. had not been characterized to date.

Compared to the OVA group, the concentrations of acetate, propionate, and lactate were increased in the fructan-fed groups. SCFAs and lactate act as a bridge between the microbiota and the immune system to maintain immune homeostasis [27]. Evidence has accumulated that SCFAs affect numerous cell types, including dendritic cells, T cells and macrophages, through G-protein-coupled receptor (GPCR) activation. Acetate (C2, 10 mM), propionate (C3, 0.5 mM), and butyrate (C4, 0.5 mM) favored the upregulation of IL-10 in Th1 cells with regulatory functions via GPR43 [28, 29], and lactate has been shown to activate GPR81 [30]. In a mouse model of corneal infection, propionate feeding (500 mM over 3 weeks) was found to have immunomodulatory effects at ocular lesion sites by concomitantly increasing Treg representation and reducing Th1 and Th17 pro-inflammatory T cells [31]. In murine and human macrophages stimulated with lipopolysaccharides, exogenous lactate administration inhibited Toll-like receptor 4-dependent pro-inflammatory responses [32], and deletion of GPR81 gene in mice led to increased Th1/Th17 cell differentiation and reduced Treg cell differentiation due to decreased expression of immune-regulatory factors such as IL-10 [33].

**Fig. 7:**
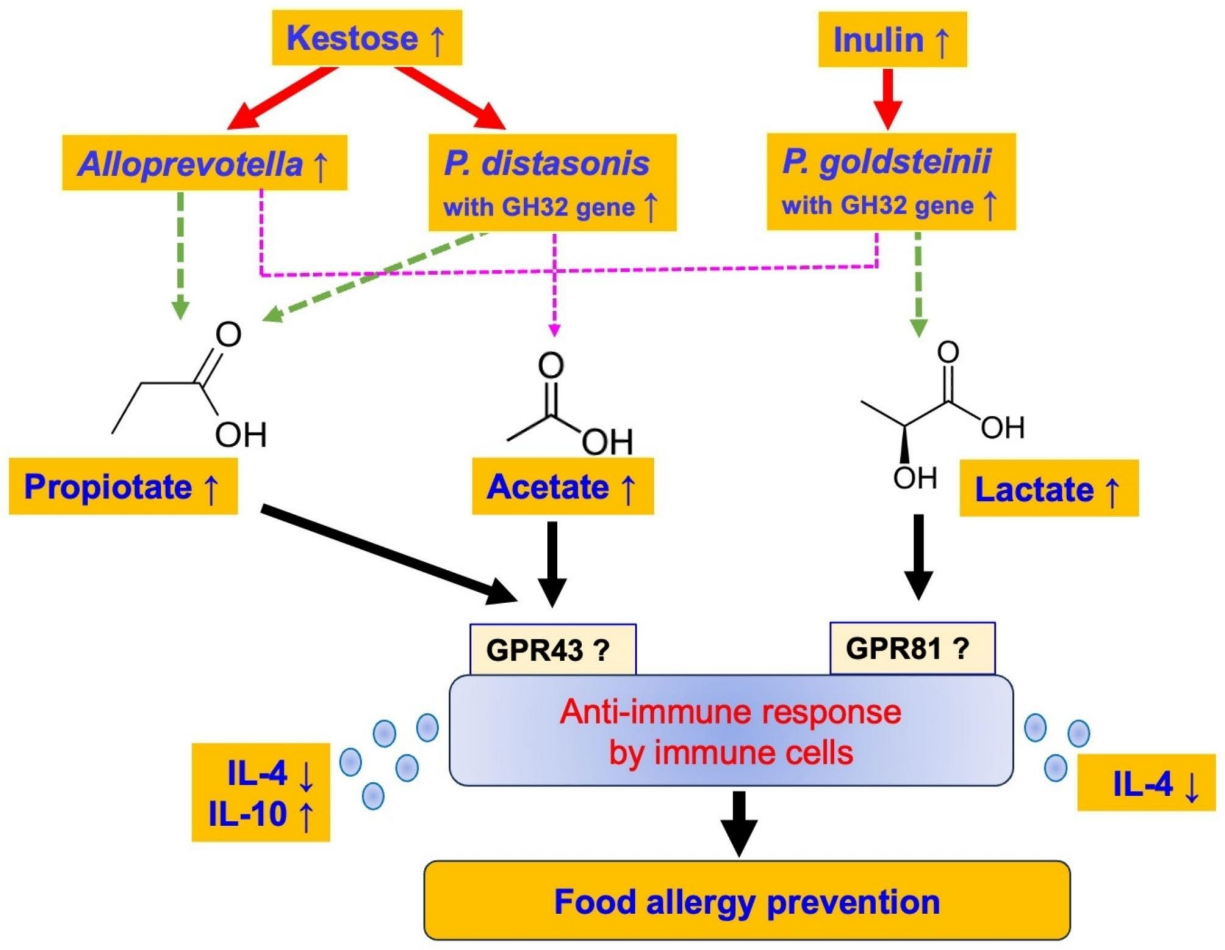
Proposed mechanism of OVA-induced food allergy prevention by fructan administration. Blue letters indicate the results of this study. Solid red lines indicate effects shown in this study, and dashed lines indicate possible effects suggested by this study. Solid black lines indicate effects suggested by previous studies

As shown in Fig. 7, the low-DP Kes and high-DP Inu may regulate food allergy via different pathways in the OVA-allergic mouse model. Kes may significantly increase the relative abundances of *Alloprevotella* and *P. distasonis* having GH32 with Kes-degrading ability, resulting in a significant increase in the intestinal propionate concentration and inducing anti-immune responses probably via propionate-specific receptors. In contrast, Inu may significantly increase the relative abundance of *P. goldsteinii* having GH32 with Inu-degrading ability, resulting in a significant increase in the intestinal lactate concentration and inducing anti-immune responses probably via lactate-specific receptors. Kes + Inu may also be involved in acetate-mediated anti-immune responses.

The present study has a few limitations. The effects on immune cells were not clarified in detail, the mechanism of allergic inhibition through SCFAs or GPCR was not fully verified, only OVA was used as an allergen, only Bacteroidota reduced by OVA allergy sensitization was focused and Firmicutes increased on the contrary was not analyzed, and a more comprehensive analysis of the changes in the gut microbiota induced by OVA and fructan was insufficient. In addition to addressing these issues, in future, we will conduct ingestion studies of *P. distasonis* and *P. goldsteinii* in germ-free mice and intervention studies in infants to establish a method to prevent food allergy through the intake of fructans.

## Conclusion

Combined intake of the low-DP Kes and high-DP Inu suppressed allergy scores more effectively than single intake and Kes and Inu likely have different allergy prevention mechanisms, suggesting that the combined intake of short and long fructans may have an allergy-preventive effect.

## Methods

### Mice

In total, 28 5-week-old female BALB/c mice weighing 14–19 g were purchased from SLC Japan (Hamamatsu, Japan). The mice were maintained under a conventional environment in appropriate numbers in cages (n = 2 or 3 /cage) lined with soft chips at 24 ± 2 °C with a 12-h light/dark cycle, with air flow and *ad libitum* access to food and water. Experimental diets were purified into pellets by CLEA Japan (Tokyo, Japan). The cages were placed randomly avoid the influence of environmental factors on the responses of mice. All animal studies were conducted following the ARRIVE guidelines and the Animal Experimentation Guidelines of Nagoya University of Arts and Sciences. The study was approved by the ethics committee of the Laboratory Animal Care Committee of Nagoya University of Arts and Sciences, in accordance with the standards of the Ministry of the Environment (approval No. 128).

### Outline of experiment

**Fig. 8:**
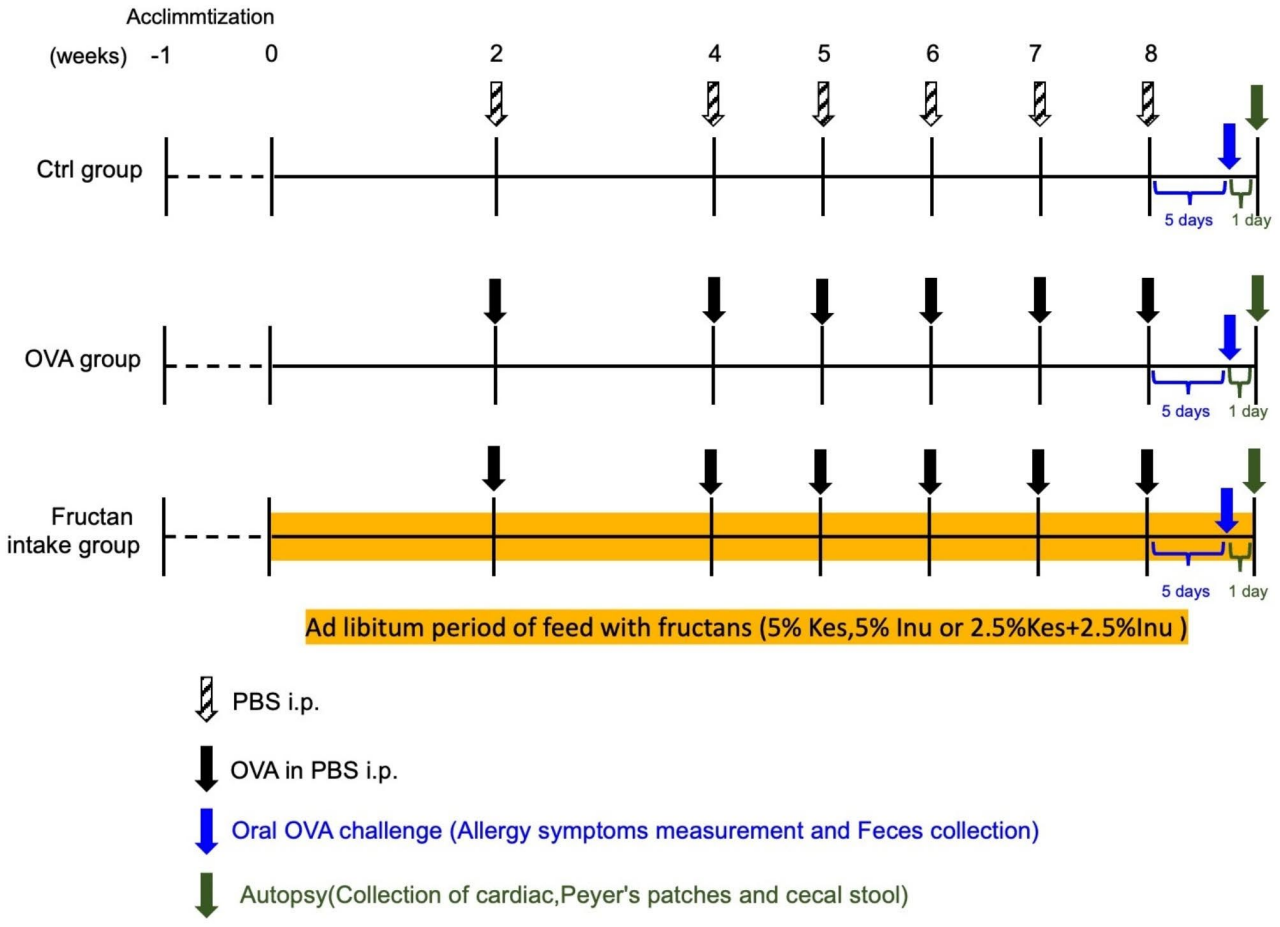
Experimental schedule for OVA-induced food allergy mouse model establishment

Figure 8 illustrates the outline of the experiment. In this study, we used a model in which Balb/c mice, which are commonly used in allergy studies, were sensitized by intraperitoneal administration [34]. All mice were divided into a control group (Ctrl group; n = 5), OVA-induced allergy group (OVA group; n = 5), OVA-induced allergy and Kes dose group (Kes group; n = 6), OVA-induced allergy and Inu dose group (Inu group; n = 6), and OVA-induced allergy and Kes and Inu dose group (Kes + Inu group; n = 6). Mice were randomly grouped using Excel and two or three mice were kept in a cage. After 1 week of pre-rearing with CLEA Rodent Diet CE-2 (CLEA Japan), experimental diets were fed from 0 week (before intraperitoneal OVA administration) until autopsy was conducted for primary prevention of OVA allergy. The control diet included AIN-93G semi-synthetic feed (CLEA Japan), whereas the composition of the diets for the experimental groups was based on AIN-93G but included some changes. In the Kes, Inu, and Kes + Inu groups, 5% cellulose was replaced with 5% Kes (Itochu Sugar, Hekinan, Japan; >97% purity), 5% Inu (Inulia®; Teijin, Tokyo, Japan: DP = 8–13), and 2.5% Kes + 2.5% Inu, respectively. Mice were immunized six times between weeks 2 and 8, following which they were assessed for symptom reduction after challenging with OVA and their fecal samples were collected. Mice in the Ctrl group received intraperitoneal phosphate-buffered saline (PBS) injections. On the day after the OVA challenge, the mice were anesthetized using isoflurane inhalation. Anesthesia was confirmed by loss of stand-up reflex and pain reflexes that occur due to stimulation of toes, tail, and ears with tweezers. Thereafter, cardiac puncture was preformed (euthanasia) and cardiac blood was collected for IgE measurements. Peyer’s patches were collected for cytokine mRNA expression analysis and cecal contents were collected for microflora analysis and SCFA measurements. Serum was stored at–20 °C, and cecal contents and organs were stored at − 80 °C until further use.

### Induction of food allergy in mice

Mice in all groups except the Ctrl group were intraperitoneally immunized with 100 µg OVA and 100 µL of aluminum hydroxide dissolved in PBS on the first day of week 2 and 50 µg OVA and 50 µL of aluminum hydroxide dissolved in PBS on the first day of weeks 4–8. Mice in the Ctrl group received intraperitoneal PBS injections of the same amount.

### Preparation of OVA

OVA used for the sensitization of mice was isolated and purified from commercial egg albumen using a combination of ammonium sulfate precipitation, sulfuric acid, and isoelectric precipitation methods. The purity was confirmed as a single band on sodium dodecyl sulfate-polyacrylamide gel electrophoresis.

### Evaluation of allergic symptoms

Five days after OVA sensitization in week 8, all groups were challenged orally with 50 mg OVA in 200 µL of PBS. Immediately after the administration, allergy scores were measured by several researchers in a blind test for 30 min, with the maximum score based on the allergic symptom. Allergic symptoms were scored as follows: 0, no symptoms; 1, scratching around the nose and head, an unusual behavior that is distinctly different from normal behavior; 2, swelling around the eyes and mouth; 3, wheezing, difficult respiration, cyanosis around the mouth and tail; 4, no activity after stimulation or tremors and convulsions; and 5, death [35]. Rectal temperature was measured using a thermometer before and 30 min after oral administration of OVA, and the change between the two was calculated. The Humane endpoint was defined as a decrease in rectum temperature of at least 3℃ degrees. However, no applicable mice in this experiment.

### ELISA

For OVA-specific IgE, OVA solution (1 mg/mL PBS) was solidified in 96-well microplates (Nunc-Immuno™ Plate; Thermo Fisher Scientific, Waltham, MA, USA) at 4 °C overnight. After blocking with 1% skim milk/PBS with 0.05% Tween-20 at 37 °C for 1 h, serum (1:9 PBS) was added and the plate was incubated at 37 °C for 1 h. Then, POD-linked anti-mouse IgE (GAM/IgE(Fc)/PO; 1:1,000 PBS; Nordic Immunology Laboratories, Tilburg, Netherlands) was added and the plate was incubated at 37 °C for 1 h. Color was developed using tetramethylbenzidine (TMB) and the reaction was stopped by adding 1 N HCl. Absorbance at 450 nm was measured using a microplate reader (Corona Electric, Hitachinaka, Japan).

For OVA-specific IgA, an IgA extraction solution was made by dissolving one protease inhibitor capsule tablet (lot No.: 56,079,200; Sigma-Aldrich, St. Louis, MO, USA) in 2 mL of sterile PBS followed by 25-fold dilution. One milliliter of the extraction solution was added to 0.1 g of collected feces and stirred at 4 °C overnight. After 10 min of centrifugation (4 °C, 15,000 rpm), the supernatant was collected to prepare the sample solution. OVA solution (1 mg/mL PBS) was solidified in 96-well microplates at 4 °C overnight. After blocking with protein-free blocking buffer at 37 °C for 1 h, the sample solution was added and the plate was incubated at 37 °C for 1 h. Then, POD-linked anti-mouse IgA (Lot No. 60,203,562; 1:5,000 PBS; Zymed Laboratories, South San Francisco, CA, USA) was added and the plate was incubated at 37 °C for 1 h. Color was developed using TMB and the reaction was stopped by adding 1 N HCl. Absorbance at 450 nm was measured using the microplate reader.

For total IgA, anti-mouse IgA (α-chain specific) antibody (M1272; Bethyl Laboratories, Montgomery, TX, USA) (1 mg/1 mL PBS) was added to a 96-well microplate and incubated at 4 °C for 1 h. After blocking with protein-free blocking buffer at 37 °C for 1 h, sample solution (1:9 PBS) was added and the plate was incubated at 37 °C for 1 h. Then, POD-linked anti-mouse IgA (Lot No. 60,203,562; 1:5,000 PBS; Zymed) was added and the plate was incubated at 37 °C for 1 h. Color was developed using TMB and the reaction was stopped by adding 1 N HCl. Absorbance at 450 nm was measured using the microplate reader.

### RNA extraction and quantitative reverse transcription PCR (RT-qPCR)

RNA was extracted from Peyer’s patches using a BioMasher® II Micro Tissue Homogenizer (DWK Life Sciences, Millville, NJ, USA) and RNeasy® Mini Kit (Qiagen, Hilden, Germany). RNA concentrations were measured using the Qubit™ RNA (Broad-Range) Assay Kit (Invitrogen, Carlsbad, CA, USA). The RNA was reverse transcribed using the High-Capacity RNA-to-cDNA Kit (Thermo Fisher Scientific). qPCRs were run using PowerUp SYBR Green Master Mix (Thermo Fisher Scientific) and primers targeting the IL-2, IL-4, IL-6, and IL-10 genes. The 18 S rRNA gene was used as a reference gene. Primer sequences are listed in Additional file 1. The amplification cycle protocol consisted of holding at 95 °C for 2 min followed by 50 cycles of 10 s at 95 °C, 60 s at 60 °C, and 1 min at 72 °C. After amplification, melting curve analysis was performed to confirm the PCR products. Quantification was performed using the ΔΔCt method.

### 16 S rRNA gene sequencing

Genomic DNA was extracted from feces as previously reported [36]. Briefly, frozen fecal samples were thawed on ice, and 100 mg of each sample was suspended in a solution containing 4 M guanidium thiocyanate, 100 mM Tris-HCl (pH 9.0), and 40 mM ethylenediaminetetraacetic acid. The samples were disrupted using zirconia beads in a FastPrep FP100A instrument (MP Biomedicals, Santa Ana, CA, USA). DNA was extracted from the bead-treated suspensions using a Magtration System 12GC and GC series MagDEA DNA 200 (Precision System Science, Matsudo, Japan). For PCR amplification of the V3-V4 region of prokaryote 16 S rRNA genes, the Pro341F and Pro805R primers were used (Table S1). Sequencing was conducted at Bioengineering Lab (Sagamihara, Japan). Paired-end sequencing (2 × 300 bp) was performed using the Illumina MiSeq platform (Illumina, San Diego, CA, USA) and a MiSeq Reagent Kit v3 (Illumina).

### Bioinformatics analysis

QIIME2 (v2022.2) was used for 16 S rRNA gene sequence analysis [37]. Quality filtering and denoising of the sequence data were conducted using the DADA2 pipeline (parameters: p-trunc-len-f 290 and p-trunc-len-r 190) [38]. The filtered output sequences were assigned to taxa using the “qiime feature-classifier classify-sklearn” command, employing default parameters [39]. Greengenes2 (v2022.10) was used as the reference database for taxonomic assignments [40]. Alpha diversity was calculated, and principal coordinate analysis of variance with weighted UniFrac distances was performed using the “qiime diversity core-metrics-phylogenetic” command. Beta diversity was assessed with weighted UniFrac distances and the “qiime diversity beta-group-significance” command.

### Quantitative analysis of intestinal microorganisms using qPCR

We aligned the nucleotide sequences of GH family 32 genes and their homologs in *P. distasonis* listed in the Carbohydrate-Active enZYmes Database (Additional file 2), and a primer set was designed to amplify the GH32 gene fragment of *P. distasonis* from the consensus sequence (Additional file 1). *P. goldsteinii* BFG-241 was the only *P. goldsteinii* strain with GH32 listed in the Carbohydrate-Active enZYmes Database. Sequences of *P. goldsteinii* BFG-241 and GH32 homologs from *P. goldsteinii* strain MTS01 and a closely related strain, *Bacteroides fragilis*, were aligned (Additional file 3). A primer set was designed to amplify the *GH32* gene fragment of *P. goldsteinii* from a sequence common to *P. goldsteinii* and distinct from *B. fragilis* (Additional file 1).

To quantify GH32 gene copy numbers in fecal DNA samples, qPCR was conducted using the QIAcuity One 5plex Platform System FUL-1 (Qiagen). Each reaction mixture (16.875 µL) contained 5.500 µL of 3× EvaGreen PCR Master Mix (Qiagen), 0.132 µL each of forward and reverse primers (50 µM), 9.611 µL of RNase-free water, and 1.500 µL of each fecal DNA. The primers used to detect the GH32 gene are listed in Additional file 1. The PCR mixture was pipetted into each of the inlet ports of a QIAcuity Nanoplate 8k with 96 wells (Qiagen), which was then sealed with a QIAcuity Nanoplate Seal (Qiagen) using a roller. The plate was placed in the instrument, and the priming, cycling, and imaging steps were performed automatically. The amplification cycle protocol consisted of an initial denaturation at 95 °C for 2 min, followed by 40 cycles of denaturation at 95 °C for 15 s, annealing at 60 °C for 15 s, and extension at 72 °C for 15 s, and a final extension at 72 °C for 1 min. Image acquisition was performed using a 150-ms exposure time.

The data were analyzed using the QIAcuity 2.0.20 software suite (Qiagen), which automatically calculates and defines the optimal threshold for discriminating between positive and negative partitions. GH32 gene copy numbers in each sample were normalized to the total number of bacteria. Specifically, the GH32 gene copy number was defined as the copy numbers of GH32 genes per microliter of template DNA divided by the total number of bacteria per microliter of template DNA. The total number of bacteria was estimated by quantifying the copy numbers of 16 S ribosomal RNA genes by qPCR using QuantStudio 3 (Thermo Fisher Scientific). The reaction solution was prepared for each template DNA using the PowerTrack SYBR Green Master Mix (Thermo Fisher Scientific) according to the manufacturer’s instructions. The primers used to detect the 16 S rRNA gene are listed in Additional file 1. The amplification cycle protocol consisted of initial denaturation at 95 °C for 2 min, followed by 40 cycles of denaturation at 95 °C for 10 s, annealing at 60 °C for 15 s, extension at 72 °C for 15 s, and a final extension at 72 °C for 1 min. After amplification, melting curve analysis was performed to confirm the specificity of the PCR products. Absolute quantification was performed using a PCR fragment of the 16 S RNA gene from *P. distasonis* JCM 5825.

### Enzymatic reaction assays using GH32-expressing *E. coli*

Extracellular GH32 enzyme activity of *P. distasonis* and *P. goldsteinii* was examined as previously reported [25]. DNA isolated from fecal samples from the Kes and Inu groups was used as template DNA for the GH32 genes of *P. distasonis* and *P. goldsteinii*, respectively. The genes were amplified using the KOD Plus DNA polymerase kit (Toyobo, Osaka, Japan) and primers listed in Additional file 1, and fused using the In-Fusion Cloning kit (Takara Bio, Kusatsu, Japan) according to the manufacturer’s instructions. pCDF-PgsA was used for gene cloning. This plasmid was developed to study the activities of extracellular GH32 enzymes on *E. coli* cells using the surface display system [18]. *E. coli* JM109 (Takara Bio) cells were transformed with the fused plasmids, which were extracted from transformed cells using the FastGene Plasmid Mini Kit (Nippon Genetics, Tokyo, Japan) and used to transform *E. coli* BL21 (DE3, Takara Bio). Transformed *E. coli* BL21 (DE3) cells were cultured in Terrific broth (Sigma-Aldrich) supplemented with 12.5 µg/mL of streptomycin sulfate at 30 °C for 24 h. Transformed *E. coli* BL21 (DE3) cells were cultured in Overnight Express™ Instant TB Medium (Merck, Darmstadt, Germany) supplemented with antibiotics at 24 °C for 24 h. Then, 1.0 mL of the culture was transferred into an Eppendorf tube and centrifuged at 6,000 rpm for 3 min to collect recombinant *E. coli* cells, which were suspended in 0.5 mL of 10 mM PBS (pH 7.0) to prepare 2× cell solutions. To 360 µL of 10 mM PBS (pH 7.0) containing 5% (w/w) of each of sucrose (Fujifilm-Wako, Osaka, Japan), Kes, and Inu from chicory (Sigma-Aldrich), 40 µL of each 2× cell solution was added, and the conversion reaction was performed at 37 °C for 30 min. The reaction vessel was a 96 Well Deep Well Plate (AxyGen Scientific, Union City, CA, USA), and the incubator was an MBR-034P shaker (Taitec, Nagoya, Japan). After the reaction, 180 µL of the reaction solution was sampled, and the centrifugal supernatant was incubated at 90 °C for 5 min to stop the conversion reaction. After dilution by adding 900 µL of water to 100 µL of the reaction solution, the solution was filtered through a 0.45-µm pore filter and used for high-performance liquid chromatography (HPLC). Chromatographic separations were performed using an HPLC Prominence (Shimadzu, Kyoto, Japan) using tandemly coupled Shodex Sugar KS802 (8.0 mm I.D. × 300 mm) and Shodex Sugar KS801 (8.0 mm I.D. × 300 mm) columns (Showa Denko, Tokyo, Japan). The mobile phase was 1 mM PBS (pH 7.0). The elution program consisted of 20 min with a flow rate of 0.25 mL/min, and the column temperature was maintained at 80 °C. The eluents were monitored using RID-20 A (Shimadzu). The Kes- and Inu-degrading activity of GH32 gene-expressing *E. coli* was evaluated as relative degradation activity, which is the ratio (%) of fructose production when Kes or Inu is used as a substrate to sucrose production.

### Determination of SCFA and lactate concentrations in cecal contents samples

The carboxyl group of SCFAs (acetate, propionate, and butylate) and lactate in each fecal sample was labeled with 2-nitrophenyl hydrazide using a Short- and Long-Chain Fatty Acid Analysis Kit (YMC, Kyoto, Japan) with the improved kit protocol. Briefly, 20–50 mg fecal sample was weighed into a 1.5-mL Eppendorf tube. One milliliter of PBS was added to the tube on ice, and the tube was vortexed for 30 s. The tube was centrifuged at 15,000 rpm at 4 °C for 2 min. The supernatant was used for the labeling reaction. Fifty microliters of supernatant was mixed with 50 µL of PBS, 200 µL of 2 mM caproic acid (Fujifilm-Wako) as an internal standard, 200 µL of 20 mM 2-nitrophenylhydrazin (in water), and 200 µL of 0.25 M N-(3-dimethylaminopropyl)-N′-ethylcarbodiimide hydrochloride HCl [in ethanol, with an equal volume of 3% pyridine in ethanol (v:v)]. The mixture was heated at 60 °C for 20 min. Then, 200 µL of 15% (w/v) potassium hydroxide was added, and the mixture was incubated at 60 °C for 15 min. The reaction mixture was added to 4 mL of a 3.8:0.4 (v:v) mixture of 0.03 M PBS (pH 6.4):0.5 M hydrochloric acid and then filtered through a 0.45-µm filter (PTFE, Puradisc™ 13 mm Syringe Filters, Whatman, Kent, England). The SCFA derivatives were extracted using 5 mL of diethyl ether. The diethyl ether layer was evaporated to dryness under a nitrogen stream at room temperature. The residue was dissolved in 200 µL of methanol, and 10 µL of each sample was analyzed by HPLC to quantify the major SCFAs. Chromatographic separations were performed using an HPLC Nexera XL series HPLC Instrument (Shimadzu) and a YMC-Pack FA HPLC column (6.0 mm I.D. × 250 mm; YMC). The solvent system for elution from the YMC-Pack FA column consisted of 30:16:54 (v:v:v) acetonitrile (Fujifilm-Wako):methyl alcohol (Fujifilm-Wako):ultrapure water. The elution program consisted of 20 min with a flow rate of 1.2 mL/min, and the column temperature was maintained at 50 °C. The eluents were monitored at 400 nm to detect derivatized 2-nitrophenylhydrazin.

### Statistical analysis

Alpha diversity results were analyzed using the Kruskal–Wallis test followed by Dunn’s multiple comparisons test in GraphPad Prism v9.5.1 (GraphPad Software, San Diego, CA, USA). Beta diversity was assessed, and *P*-values were calculated using permutational multivariate analysis of variance. Statistical analyses of microbiota were performed using the nonparametric Mann–Whitney test or Kruskal–Wallis test, and all other statistical analyses were performed using parametric ordinary one-way ANOVA in GraphPad Prism v.9.5.1. Statistical significance was set at *P* < 0.05.

### Electronic supplementary material

Below is the link to the electronic supplementary material.

Supplementary Material 1

Supplementary Material 2

Supplementary Material 3

## Data Availability

The GH32 gene sequences of *P. distasonis* and *P. goldsteinii* obtained in this study are available in GenBank (GenBank accession No. LC771243 and LC771244, respectively).

## Abbreviations

DP: Degree of polymerization
ELISA: Enzyme-linked immunosorbent assay
GH: Glycoside hydrolase
HPLC: High-performance liquid chromatography
Inu: Inulin
Kes: 1-kestose
OVA: Ovalbumin
PBS: Phosphate-buffered saline
RT-qPCR: Quantitative reverse transcription PCR
SCFA: Short-chain fatty acid
