# Supplementary material for: Combined oral intake of short and long fructans alters the gut microbiota in food allergy model mice and contributes to food allergy prevention
Source: BMC Microbiol. 2023 Sep 22;23:266. doi: 10.1186/s12866-023-03021-6 (PMC10515425; doi:10.1186/s12866-023-03021-6)
Supplement: Supplementary file 2 — Supplementary Material 2 [file 12866_2023_3021_MOESM2_ESM.pptx]

## Slide 1
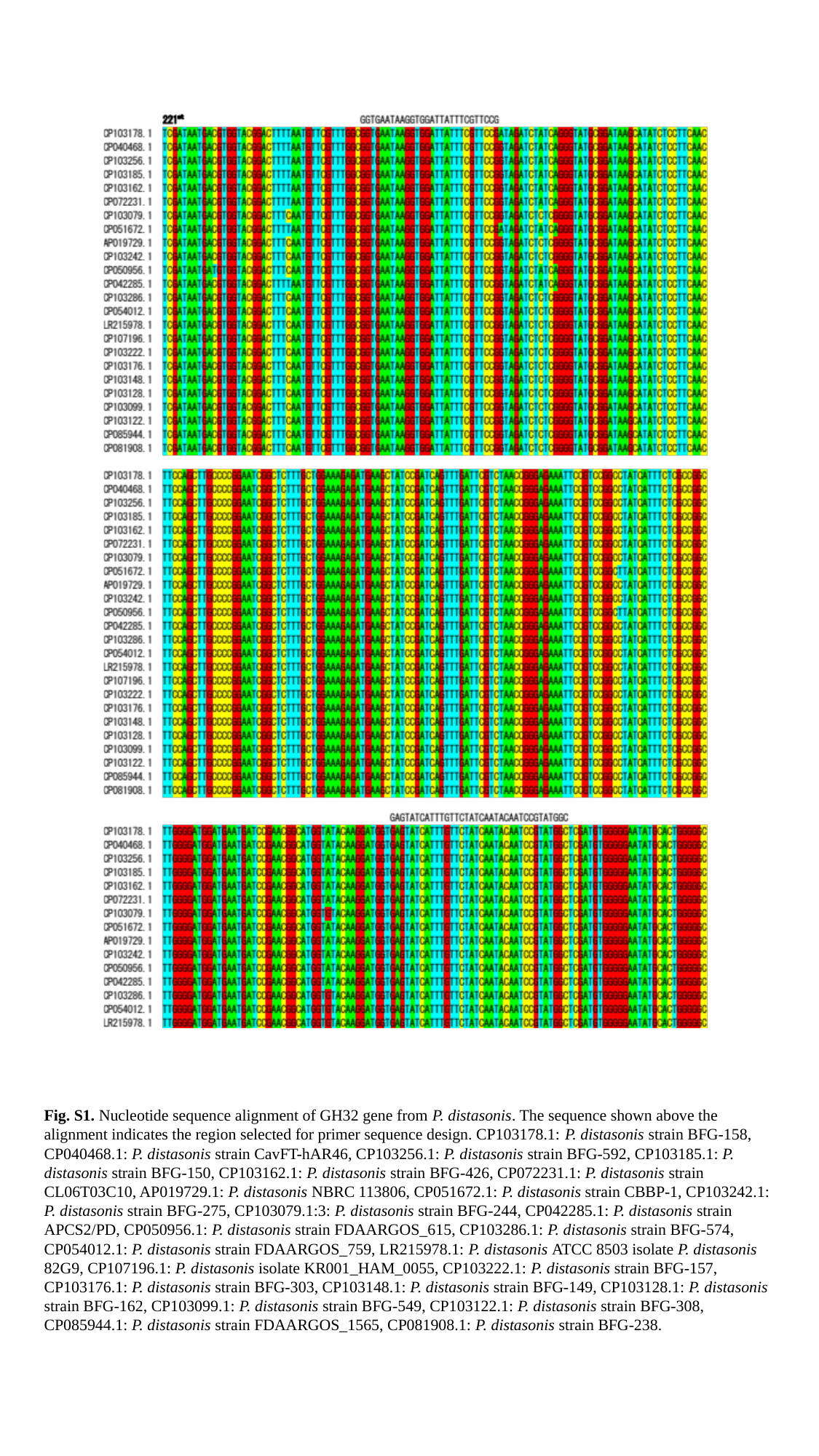

Fig. S1. Nucleotide sequence alignment of GH32 gene from P. distasonis. The sequence shown above the alignment indicates the region selected for primer sequence design. CP103178.1: P. distasonis strain BFG-158, CP040468.1: P. distasonis strain CavFT-hAR46, CP103256.1: P. distasonis strain BFG-592, CP103185.1: P. distasonis strain BFG-150, CP103162.1: P. distasonis strain BFG-426, CP072231.1: P. distasonis strain CL06T03C10, AP019729.1: P. distasonis NBRC 113806, CP051672.1: P. distasonis strain CBBP-1, CP103242.1: P. distasonis strain BFG-275, CP103079.1:3: P. distasonis strain BFG-244, CP042285.1: P. distasonis strain APCS2/PD, CP050956.1: P. distasonis strain FDAARGOS_615, CP103286.1: P. distasonis strain BFG-574, CP054012.1: P. distasonis strain FDAARGOS_759, LR215978.1: P. distasonis ATCC 8503 isolate P. distasonis 82G9, CP107196.1: P. distasonis isolate KR001_HAM_0055, CP103222.1: P. distasonis strain BFG-157, CP103176.1: P. distasonis strain BFG-303, CP103148.1: P. distasonis strain BFG-149, CP103128.1: P. distasonis strain BFG-162, CP103099.1: P. distasonis strain BFG-549, CP103122.1: P. distasonis strain BFG-308, CP085944.1: P. distasonis strain FDAARGOS_1565, CP081908.1: P. distasonis strain BFG-238.
