# Supplementary material for: Combined oral intake of short and long fructans alters the gut microbiota in food allergy model mice and contributes to food allergy prevention
Source: BMC Microbiol. 2023 Sep 22;23:266. doi: 10.1186/s12866-023-03021-6 (PMC10515425; doi:10.1186/s12866-023-03021-6)
Supplement: Supplementary file 3 — Supplementary Material 3 [file 12866_2023_3021_MOESM3_ESM.pptx]

## Slide 1
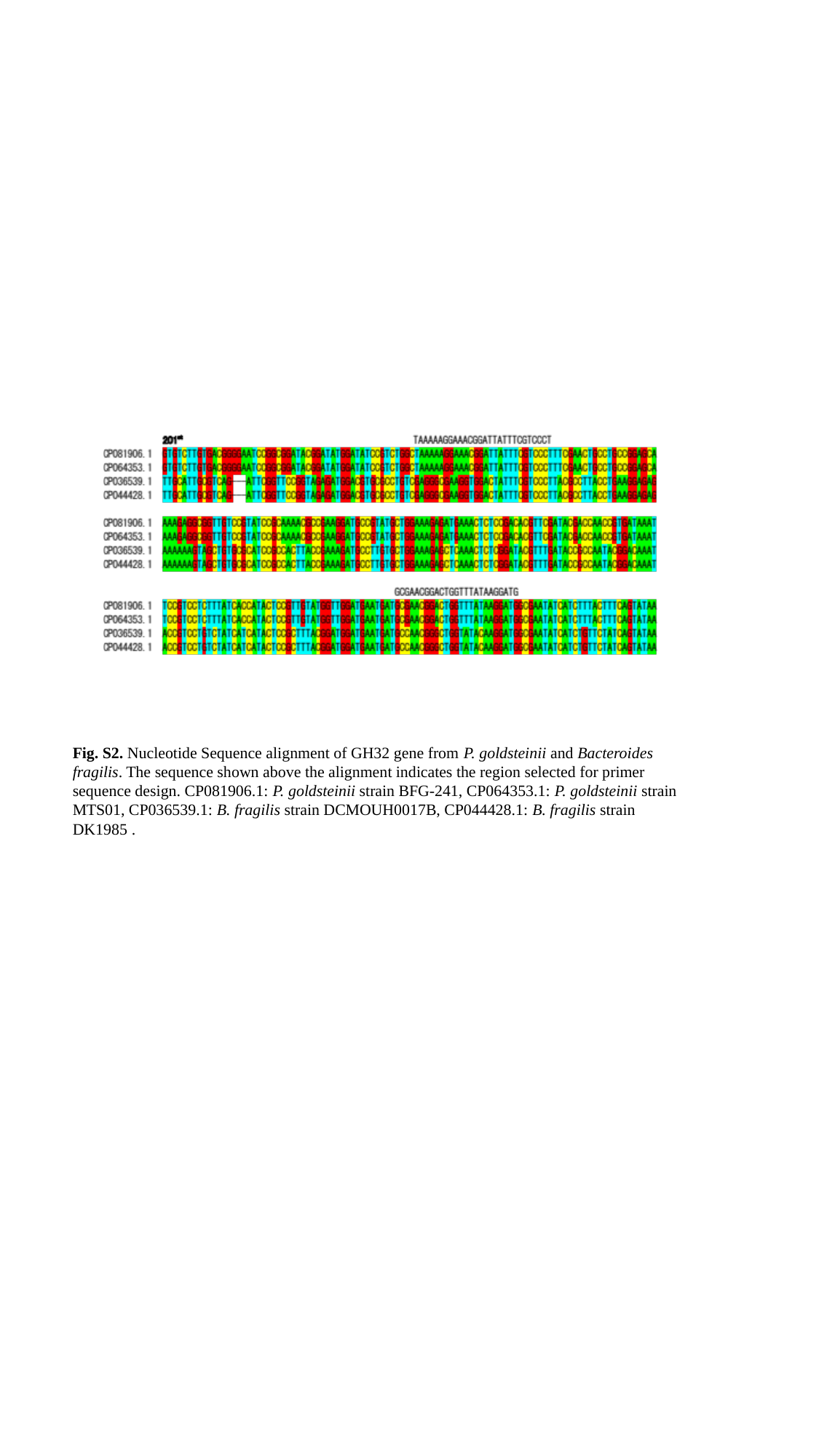

Fig. S2. Nucleotide Sequence alignment of GH32 gene from P. goldsteinii and Bacteroides fragilis. The sequence shown above the alignment indicates the region selected for primer sequence design. CP081906.1: P. goldsteinii strain BFG-241, CP064353.1: P. goldsteinii strain MTS01, CP036539.1: B. fragilis strain DCMOUH0017B, CP044428.1: B. fragilis strain DK1985 .
